# Paratesticular liposarcoma: a case report

**DOI:** 10.11604/pamj.2019.33.282.19545

**Published:** 2019-08-02

**Authors:** Karima Mouden, Soukaina Wakrim, Afaf Semmar

**Affiliations:** 1 Department of Radiation Oncology, National Oncology Institute, Rabat, Morocco; 2 Department of Radiology, Ibn Zohr University, Faculty of Medicine and Pharmacy, Agadir, Morocco

**Keywords:** Paratesticular liposarcoma, giant liposarcoma, well-differentiated liposarcoma, pelvic tumor

## Abstract

Paratesticular liposarcomas (PLS) is a very uncommon pathology type of paratesticular sarcomas, with less 200 similar cases reported to date in the English literature. There are a few cases regarding giant paratesticular liposarcoma measuring over 10 cm. We present an unusual case with a giant well differentiated PLS of the left testis extended to the pelvic cavity. We report the case of a 55-year-old man who presented with large left groin mass. The patient underwent left orchiectomy following a cure of a scrotal hernia. Histological and immunohistochemical findings were suggestive of a well-differentiated liposarcoma of spermatic cord. The surgical margins were positive. Metastatic work-up, which included CT of the thorax, abdomen and pelvis, did not reveal any distant metastasis in thorax but there is a left pathological external iliac lymph nodes and a left lateroplevic lipomatous mass extended to the iliac fossa and left parietocolic gutter up to the umbilicus measuring 15x7x17 cm. Our patient refused treatment. A review of the literature revealed that there are fewer cases of giant well differentiated paratesticular liposarcoma extended to the pelvic cavity were reported. This study focuses on the clinical characteristics and treatment of this rare type of tumours.

## Introduction

Paratesticular liposarcomas (PLS) are rare variant of genitourinary malignancies, occurring more often in the spermatic cord where it accounts for less than 12% of all liposarcomas [1, 2]. About 200 paratesticular liposarcomas were reported to date in the English literature [3]. Due to the scarcity of cases, there is no consensus on the best PLS treatment, and this entity is still challenging clinicians. In this paper, we report a case of a giant well-differentiated paratesticular liposarcoma of the left testis with a left pelvic mass measuring 15x7x17 cm. We present this case because giant PLS is more rare with only a few cases having been reported [4, 5].

## Patient and observation

**Figure 1:**
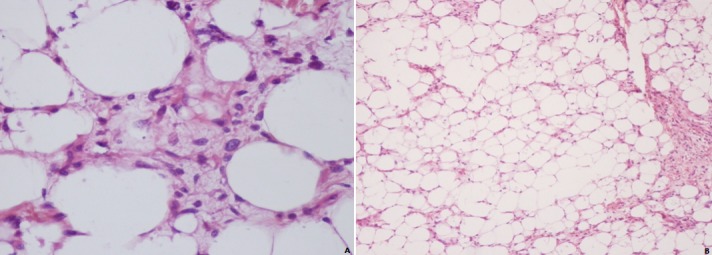
A) hematoxylin and eosin (H&E) staining showing lipoblasts with atypical nucleus indented on its surfaces by fat vacuoles; B) stromal cells with hyperchromatic nuclei

**Figure 2:**
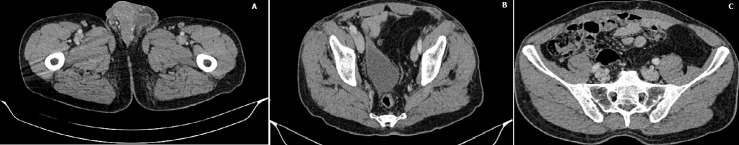
A) computed tomography showed a mass arising from groin region; B) computed tomography showed a left lateroplevic lipomatous mass extended to the iliac fossa and left parietocolic gutter measuring 15x7x17 cm repressing the bladder, sigmoid, left colon, some ileal loops and encompassing the left external iliac pedicle; C) computed tomography showed a lipomatous mass extended to the umbilicus repressing the left colon, some ileal loops and encompassing the left external iliac pedicle

A previously healthy 55-year-old man presented with an increasing scrotal swelling lasting for 2-years. Examination revealed a left hard hemiscrotal mass, distinct from the left testis, measuring approximately 15 cm × 12.5 cm. A scrotal exploration was performed under general anaesthetic where a large left groin mass was found separate to the testis; however, with suspicions for underlying mass, a left orchidectomy was performed. Microscopically, the tumor had lipoblasts with atypical nucleus indented on its surfaces by fat vacuoles and stromal cells with hyperchromatic nuclei (Figure 1). The histopathologic diagnosis was consistent with well-differentiated spermatic cord liposarcoma with positive spermatic cord margins. Computed tomography showed a left lateroplevic lipomatous mass extended to the iliac fossa and left parietocolic gutter up to the umbilicus measuring 15x7x17 cm repressing the bladder, sigmoid, left colon, some ileal loops and encompassing the left external iliac pedicle and left pathological external iliac lymph nodes measuring 15 mm (Figure 2). A staging CT of the thorax, abdomen and pelvis was performed which was negative for metastatic disease. Following completion excision of the spermatic, hemiscrotectomy and resection of the pelvic tumor, the case was referred to a sarcoma unit, but patient refused treatment.

## Discussion

Paratesticular liposarcomas, which account for 7% to 10% of all intrascrotal tumors, have their mesodermal origin derived from adipose tissue. The most common site of origin is the spermatic cord accounting for 90% of cases [6]. There are few cases of the giant PLS reported to date in the English literature [4, 5]. Liposarcomas are the most common soft tissue sarcomas. There are five histological subtypes in liposarcoma, which include well differentiated/atypical lipomatous tumor, dedifferentiated, myxoid and pleomorphic according to the 2013 World Health Organisation (WHO) classification of tumors of soft tissue and bone [7]. Our case noticed the swelling in his inguinal region two years earlier. It was mistaken for scrotal hernia like other patients reported in review of the literature [8, 9]. The diagnosis of PLS is documented by Ultrasonography (US), Computerized Tomography (CT) and Magnetic Resonance Imaging (MRI) [10]. Due to rarity of PLS, knowledge has been limited almost exclusively to case reports. The most widely used local treatment approaches include: radical orchiectomy with high ligation of the spermatic cord at the inguinal ring [6]. Incomplete excision is associated with frequent recurrence [11-13]. Khandekar *et al*. found that the 3 year local recurrence free survival was 100% for negative margins compared with 29% for positive margins [14]. Retroperitoneal lymph node dissection should be limited to patients with only radiologically suspicious lymph nodes [13]. Indications for adjuvant radiotherapy include positive margins or less than 10 mm and when the tumor is not resectable, because local recurrence following surgery alone is very high [15-17]. Cerda *et al*. [17] reported that adjuvant radiotherapy with a total dose of 54 Gy/27 or 30 fractions were found no recurrence in median 18 months of follow up (range 6 28 months). The prognosis of PLS depends on the histological cell type. The well-differentiated type have a better prognosis, but tend to have a high incidence of local recurrence [18]. Due to the paucity of cases, the role of adjuvant systemic chemotherapy in adult with PLS is not clear. When chemotherapy is indicated, treatment with vincristine, cyclophosphamide and doxorubicin is used for high grade and metastaic LPS [19].

## Conclusion

Often misdiagnosed preoperatively, paratesticular liposarcoma must be considered in the differential diagnosis of groin mass. Regardless of tumor size, radical orchidectomy with free surgical margins is recommended in order to avoid recurrence. The role of adjuvant therapies represented by radiotherapy and chemotherapy is controversial.

## Competing interests

The authors declare no competing interests.
